# Illegal Wildlife Trade in Kathmandu Valley: A Hub of Biodiversity Crime

**DOI:** 10.1002/ece3.74303

**Published:** 2026-09-13

**Authors:** Narayan Prasad Koju, Bhim Maya Tamang

**Affiliations:** ^1^ Center for Post Graduate Studies, Nepal Engineering College Pokhara University Lalitpur Nepal; ^2^ Department of Forest and Soil Conservation Ministry of Agriculture, Forests and Environment Kathmandu Nepal

**Keywords:** biodiversity loss, CITES, conservation, illegal wildlife trade, organized crime, pangolin

## Abstract

The illegal wildlife trade (IWT) poses a major driver of global biodiversity loss and poses particular challenges in biodiversity‐rich countries located along international trafficking routes. Nepal occupies a strategic position between two of the world's largest consumer markets for wildlife products, India and China, making Kathmandu Valley an important urban center for wildlife trafficking. Despite its strategic importance, long‐term analyses of wildlife crime patterns in Kathmandu Valley remain limited. This study examines patterns and temporal trends in IWT and describes the demographic characteristics of offenders in Kathmandu Valley based on 306 wildlife crime cases recorded between 2017 and 2022. Data was collected from Divisional Forest Offices, the Wildlife Crime Control Bureau, and the Department of National Parks and Wildlife Conservation. A total of 36 animal species were documented in seizures, including 31 mammals, four reptiles, and one fish species, with five identified exotic species. The most frequently seized species were the Chinese pangolin ( *Manis pentadactyla* ), leopard ( *Panthera pardus* ), red panda ( *Ailurus fulgens* ), Himalayan black bear ( *Ursus thibetanus* ), and musk deer (*Moschus* spp.). Pangolin scales and leopard skins were the most common wildlife products confiscated. The highest number of arrests occurred in 2018, with a sharp decline in 2020 that coincided with the restrictions imposed during the COVID‐19 pandemic. Among 740 individuals detained, 98% were male and 50.1% belonged to the 20–29 age group. Most offenders originated from adjoining districts of Kathmandu Valley, particularly from the Tamang community (29.1%). This male‐biased sex ratio and the pronounced representation of young adults each departed significantly from an equal distribution (*χ* ^2^ goodness‐of‐fit tests; both *p* < 0.001), whereas the annual number of recorded cases showed no significant temporal trend across the study period (Spearman's *ρ* = 0.036, *p* = 0.939). To our knowledge, this represents one of the first multi‐year spatial–demographic analysis of illegal wildlife trade in Nepal's primary urban hub, offering exploratory insights relevant to transboundary enforcement. The findings may have implications for regional conservation policy, suggesting the need for cross‐border collaboration, targeted law enforcement, and the integration of socio‐economic development in to anti‐trafficking strategies. To effectively combat IWT and safeguard Nepal's biodiversity, a coordinated approach involving strengthened legal frameworks, enhanced surveillance, public awareness campaigns, and community‐based conservation initiatives is urgently needed.

## Graphical abstract

Spatial distribution of wildlife crime incidents in Kathmandu valley.

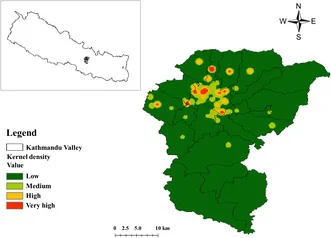

## 1 Introduction

The illegal wildlife trade (IWT) represents one of the most urgent and complex challenges in global biodiversity conservation. Defined as the unlawful harvesting, transportation, and trade of wild species and their derivatives, IWT severely undermines ecological integrity, accelerates species extinction, destabilizes local economies, and contributes to the spread of zoonotic diseases (Wyler and Sheikh 2008; Bezerra‐Santos et al. 2021). Globally, IWT is recognized as the fourth largest transnational crime, trailing only drug, arms, and human trafficking (Rosen and Smith 2010; Broussard 2016). The trade, estimated to be worth billions of dollars annually (De Greef and Raemaekers 2014), involves a wide array of flora and fauna, including mammals, reptiles, birds, and marine species, and is predominantly driven by sustained demand for traditional medicine, luxury goods, exotic pets, and bushmeat (Wyler and Sheikh 2008; Rosen and Smith 2010; Wilson‐Wilde 2010).

Asia, particularly Southeast Asia and China, functions simultaneously as both a primary market and a transit zone for many wildlife products (UNODC 2020). Wildlife commodities such as rhino horn, tiger parts, pangolin scales, and exotic reptiles are in high demand, particularly in traditional medicine markets and among consumers motivated by social status (Zhang et al. 2008). Within this regional context, Himalayan countries play a critical intermediary role. Nepal, situated between two major consumer countries, India and China, plays a dual role as a source and a strategic transit route in this illicit network. The porous borders, weak enforcement, and rugged terrain collectively facilitate the covert movement of wildlife products. Within Nepal, Kathmandu Valley stands out as a logistical and economic epicenter, making it a prominent hotspot for trafficking and trade (Nijman 2010).

Despite notable conservation successes, including zero‐poaching years for rhinos and population recovery of tigers, illegal wildlife trade continues to pose a significant threat (DNPWC 2018b; DNPWC and DFSC 2018; Mahatara et al. 2018). The involvement of organized crime syndicates further complicates enforcement, as they operate through intricate networks that span across borders (Rana and Kumar 2023). Local actors, often from socioeconomically disadvantaged backgrounds, are frequently recruited as poachers or couriers, driven by limited livelihood opportunities with limited awareness of legal repercussions and restricted access to alternative income sources (Paudel, Potter, and Phelps 2020).

Previous studies have documented the species most frequently targeted in Nepal, including the tiger ( *Panthera tigris* ), red panda ( *Ailurus fulgens* ), Chinese pangolin ( *Manis pentadactyla* ) [recently revalidated as Himalayan pangolin (*Manis aurita*) (Koju et al. 2026)], and leopard, yet few have systematically examined the temporal trends, demographic patterns of offenders, and spatial distribution of wildlife crime specifically within Kathmandu Valley (DNPWC 2018a; Paudel, Acharya, et al. 2020; Bashyal et al. 2021; Uprety et al. 2021). Understanding these dynamics is critical for tailoring conservation interventions and enforcement strategies to the unique socio‐political and geographic context of the Kathmandu Valley, which remains under‐examined.

This study addresses these gaps by analyzing 6 years of wildlife crime data from 2017 to 2022 in the Kathmandu Valley. Specifically, it (i) documents the diversity and frequency of species seized, (ii) assesses temporal trends and spatial hotspots of IWT, and (iii) describes demographic characteristics of offenders. These findings contribute to a more nuanced understanding of Nepal's role in regional IWT networks and provide empirical evidence to inform conservation planning, law enforcement prioritization, and policy formulation.

## 2 Materials and Methods

### 2.1 Study Area

This study was conducted in the Kathmandu Valley, which includes three administrative districts: Kathmandu, Lalitpur, and Bhaktapur (Figure 3). The valley covers an area of 933.73 km^2^ and is the political, economic, and cultural hub of Nepal, with a population exceeding 3 million (National Statistics Office 2021). The valley is surrounded by densely forested hills, including portions of Shivapuri Nagarjun National Park and community‐managed forests (SNNP 2017). These forested landscapes, combined with the valley's central location and access to transportation networks, make it both a source and major transit hub for illegal wildlife trade. Furthermore, the proximity to international borders and the presence of Tribhuvan International Airport enhance Kathmandu Valley's strategic importance in transboundary trafficking networks.

### 2.2 Data Sources and Collection

Primary data for this study were obtained from official wildlife crime and seizure records maintained by the Divisional Forest Offices (DFOs) of Kathmandu, Lalitpur, and Bhaktapur, the Wildlife Crime Control Bureau (WCCB), the Department of National Parks and Wildlife Conservation (DNPWC), and the Central Investigation Bureau (CIB) of the Nepal Police. Data covered a six‐years from January 2017 to December 2022. These records included detailed information on confiscated wildlife species and their derivatives, quantities seized, locations of incidents, dates of seizures, and demographic characteristics of individuals arrested.

Secondary data were compiled from published government reports, peer‐reviewed literature, and databases maintained by the International Union for Conservation of Nature (IUCN) and the Convention on International Trade in Endangered Species of Wild Fauna and Flora (CITES).

### 2.3 Data Categorization and Variables

All recorded wildlife species were taxonomically categorized and cross‐referenced with their global conservation status using the IUCN Red List and their legal protection status under CITES Appendices. Seizure records were classified based on the type of wildlife product (e.g., skin, scale, bone, live specimen), quantity confiscated, and reported methods of concealment or transportation. Offender‐related variables included gender, age group, ethnicity, and district of origin.

Spatial data for seizure locations were extracted from official records and mapped using geographic coordinates where available. In cases where GPS data were not recorded, locations were approximated based on detailed incident descriptions. Spatial analyses were conducted using ArcGIS version 13 to generate heatmaps and identify wildlife trafficking hotspots within the Kathmandu Valley. Spatial hotspot maps were generated using kernel density estimation for visualization purposes only. No inferential hotspot statistics were performed because the available data represent enforcement records rather than systematic observations.

### 2.4 Data Analysis

Descriptive statistical analyses were used to summarize the frequency, diversity, and composition of wildlife crime cases, species involved, and confiscated products. Temporal trends were assessed through annual comparisons to identify fluctuations in seizure and arrest patterns over the study period. Spatial analyses were employed to visualize the distribution of illegal wildlife trade activities and to identify high‐risk areas within the valley.

In addition to these descriptive summaries, selected inferential statistical tests were applied to determine whether specific patterns departed from chance expectation. Chi‐square goodness‐of‐fit (*χ* ^2^) tests were used to evaluate whether the sex ratio and the age‐group distribution of apprehended offenders differed significantly from an equal distribution across categories. A Spearman rank correlation was used to test for a monotonic association between calendar year and the annual number of recorded cases. Chi‐square tests of independence were used to assess whether the composition of species involved in seizures varied among years and whether offender ethnicity was associated with the species implicated in a given case. Records with missing values (this is less than 5%) for the variable under consideration were excluded from the corresponding test, and the sample size analyzed is reported alongside each result. Statistical significance was set at *α* = 0.05 for all tests.

### 2.5 Ethical Considerations

Ethical clearance for this study was obtained from the Department of Forest and Soil Conservation and the Department of Natural Resources Management, Nepal Engineering College, together with permission from the Divisional Forest Offices of Kathmandu, Lalitpur, and Bhaktapur, the Wildlife Crime Control Bureau, and the Department of National Parks and Wildlife Conservation. All personal identifiers were removed during data processing to ensure anonymity and confidentiality of individuals involved.

## 3 Results

### 3.1 Species Diversity and Seizures

A total of 306 wildlife crime cases recorded between 2017 and 2022 involved 36 species, comprising 31 mammals, four reptiles, and one fish species, including five exotic taxa. Of these, 19 species were listed under CITES Appendix I, and 34 species were classified under the IUCN Red List, including one Critically Endangered and 13 Endangered species.

The most frequently trafficked species were the Chinese pangolin ( *Manis pentadactyla* ), accounting for 22.8% of all seizure events, followed by leopard ( *Panthera pardus* ; 18.9%), red panda ( *Ailurus fulgens* ; 11.1%), Himalayan black bear ( *Ursus thibetanus* ; 5.2%), and musk deer (*Moschus* spp.; 4.90%) (Table 1). The predominance of globally threatened species highlights the conservation significance of wildlife crime occurring within the Kathmandu Valley.

**TABLE 1:** List of illegally traded species in Kathmandu Valley (2017–2022).

Common name	Scientific name	IUCN category	CITES appendices	Nationally protected	Number of seizure event
*Mammals*					
Asian elephant	*Elephas maximus*	Endangered	I	Yes	5
Barking deer	*Muntiacus vaginalis*	Least concern	NA	No	9
Black bear	*Ursus thibetanus*	Endangered	I	Yes	16
Black porcupine^a^	*Erethizon dorsatum*	Least concern	NA	No	1
Gayal^a^	*Bos frontalis*	NA	NA	No	1
Chimpanzee	*Pan troglodytes*	Endangered	I	No	1
Chinese pangolin^b^	*Manis pentadactyla*	Critically Endangered	I	Yes	70
Clouded leopard	*Neofelis nebulosi*	Endangered	I	Yes	6
Leopard	*Panthera pardus*	Vulnerable	I	No	58
Eurasian otter	*Lutralutra*	Near threatened	I	No	2
Gaur	*Bos gaurus*	Vulnerable	I	No	2
Giant flying squirrel	*Petaurista petaurista*	Least concern	NA	No	2
Blue sheep	*Pseudoisnayaur*	Least concern	NA	No	1
Himalayan tahr	*Hemitragus jemlahicus*	Near threatened	NA	No	1
Hog deer	*Axis porcinus*	Endangered	NA	No	2
Jackal	*Canis aureus*	Least concern	III	No	1
Jungle cat	*Felischaus*	Least concern	II	No	1
Langur	*Semnopithecus schistaceus*	Least concern	I	No	1
Ring tail Lemur^a^	*Lemur catta*	Endangered	I	No	1
Leopard cat	*Prionailurus bengalensis*	Vulnerable	I	No	6
Monkey	*Rhesus* spp.	Least concern	II		4
Musk deer	*Moschus* sp.	Endangered	I	Yes	15
One horned rhino	*Rhinoceros unicornis*	Endangered	I	Yes	10
Palm civet	*Paradoxurus hermaphrodites*	Least concern	III	No	2
Red panda	*Ailurus fulgens*	Endangered	I	Yes	34
Royal Bengal tiger	*Panthera tigris*	Endangered	I	Yes	10
Samber deer	*Cervus unicolor*	Vulnerable	NA	No	3
Sloth bear	*Melursus ursinus*	Endangered	I		3
Spotted deer	*Axis axis*	Least concern	NA	No	3
Striped hyena	*Hyaena hyaena*	Endangered	III	No	2
Wild boar	*Sus scrofa*	Least concern		No	4
*Reptiles*					
Bengal monitor lizard^a^	*Varanus bengalensis*	Near threatened	I	No	4
Golden monitor lizard	*Varanus flavescens*	Endangered	I	Yes	5
Mugger crocodile	*Crocodylus palustris*	Vulnerable	I	No	2
Tortoise	Unidentified	NA	NA	No	5
Birds	Multiple species				12
Fish	Unidentified				
Sea horse^a^	*Hippocampus reidi*	Near Threatened	II		1

^a^ Exotic species for Nepal.

^b^ Reported as *Manis aurita* by Koju et al. (2026).

### 3.2 Types and Quantities of Wildlife Products Seized

A diverse range of wildlife derivatives were confiscated during the study period, including skins, scales, musk pods, bile, bones, teeth, claws, and live animals. Pangolin scales constituted the largest quantity by weight, totaling 1371.57 kg. Leopard skins (*n* = 56), red panda skins (*n* = 54), and musk pods (*n* = 6) were also among the most frequently seized products (Supporting Information S1). One of the most significant individual seizures involved 162 kg of pangolin scales intercepted in 2018 at Tribhuvan International Airport, smuggled by foreign nationals, underscoring the role of international trafficking routes passing through Kathmandu Valley.

### 3.3 Temporal Trends in Seizure and Arrest Data

**FIGURE 1:**
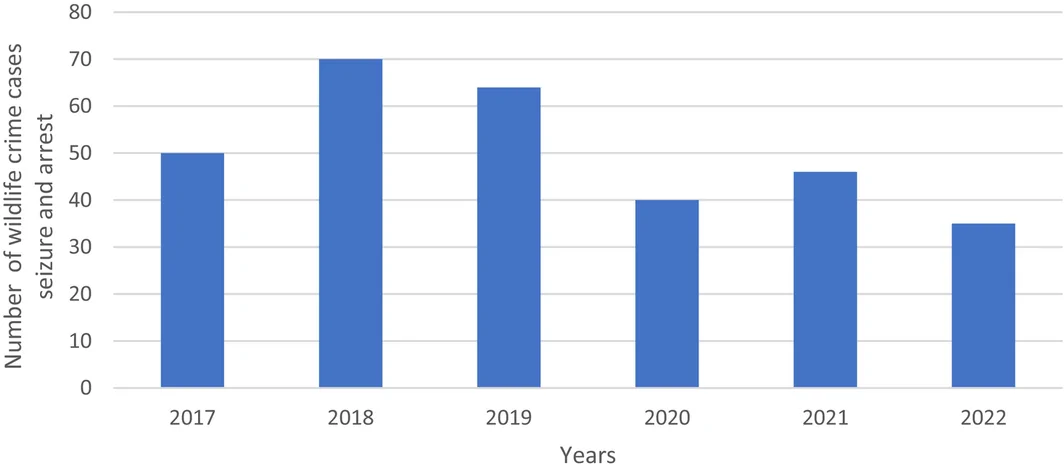
A temporal trend of wildlife crime cases.

Temporal analysis revealed marked fluctuations in wildlife crime incidents over the study period. The highest number of cases (*n* = 70) was recorded in 2018, followed by a sharp decline in 2020, coinciding with nationwide lockdowns and movement restrictions imposed during the COVID‐19 pandemic. However, seizure and arrest numbers increased again in 2021 and 2022, indicating a rapid resurgence of illegal wildlife trade activities following the relaxation of restrictions. Species‐specific trends showed a decline in seizures involving pangolins and leopards after 2019, whereas cases related to musk deer and Himalayan black bears exhibited an increasing trend over time (Figure 1; Supporting Information S1).

A Spearman rank correlation between calendar year and the annual number of recorded cases did not detect a statistically significant monotonic trend (*ρ* = 0.036, *p* = 0.939, *n* = 6 years). This indicates that the fluctuations of cases along calendar year, including the 2020 decline, do not correspond to a significant long‐term directional change. In contrast, trafficked species assemblage was not stable over the study period. A chi‐square test of independence indicated that the relative frequency of species represented in seizures varied significantly among years (*χ* ^2^ = 385.14, df = 300, *p* < 0.001), suggesting the composition of the trafficked‐species assemblage was not stable over the study period.

### 3.4 Demographic Profile of Offenders

A total of 740 individuals were apprehended in connection with wildlife crime cases during the study period. The vast majority were male (98%), with females accounting for only 2% of arrests. Offenders aged 20–29 years constituted the largest age group (50.1%), suggesting that young adults are particularly vulnerable to recruitment by trafficking networks.

This male‐biased sex ratio differed significantly from an equal (50:50) distribution (*χ* ^2^ = 498.23, df = 1, *p* < 0.001, *n* = 740), and the age‐group distribution likewise departed significantly from a uniform distribution across the five age categories (*χ* ^2^ = 453.86, df = 4, *p* < 0.001, *n* = 683 offenders with age recorded). The mean age of apprehended offenders was 30.7 years (median = 28 years; range = 14–76 years).

Ethnically, the highest proportion of offenders belonged to the Tamang community (29.1%), followed by Chhetri (14.7%) and Brahmin (14.1%). Most individuals originated from districts surrounding the Kathmandu Valley, particularly Nuwakot, Dhading, and Kavrepalanchok. In addition, 36 foreign nationals (4.8%) were implicated in wildlife crime cases, including individuals from India (*n* = 20), China (*n* = 12), Korea (*n* = 2), Australia (*n* = 1), and Pakistan (*n* = 1) (Supporting Information S1).

A chi‐square test of independence found no significant association between offender ethnicity and the wildlife species involved in the corresponding seizure case (*χ* ^2^ = 201.50, df = 176, *p* = 0.091). Although the Tamang community accounted for the largest single share of arrests, the species composition of seizures did not differ significantly among the principal ethnic groups represented among offenders.

### 3.5 Geographic Distribution and Hotspot Mapping

**FIGURE 2:**
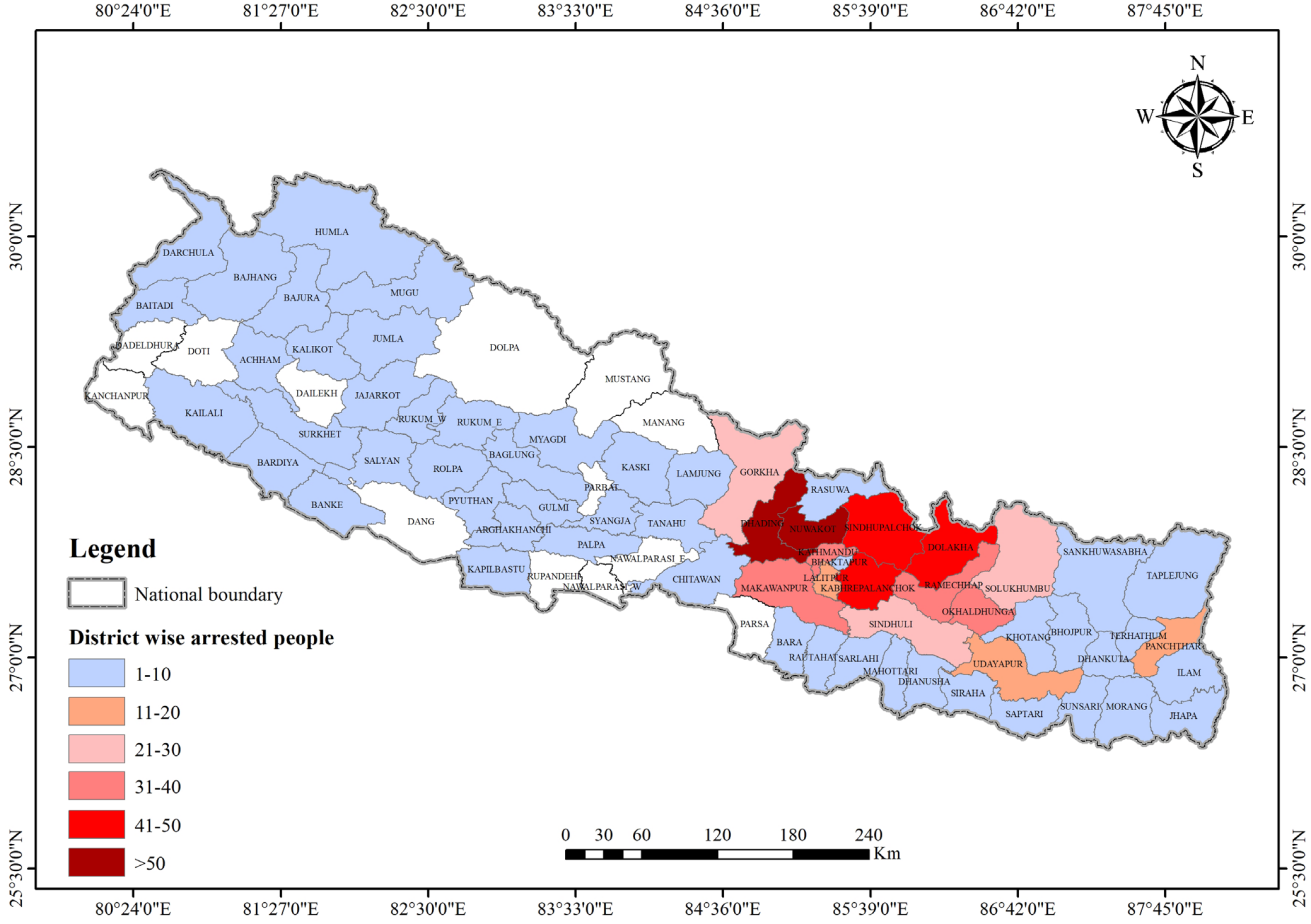
Origin of offender individuals in Nepal (2017–2022).

Offender origin data indicated participation from 64 of Nepal's 77 districts, demonstrating the extensive geographic reach of wildlife trafficking networks linked to Kathmandu Valley. The highest number of offenders originated from Nuwakot (*n* = 55), Dhading (*n* = 51), Kavrepalanchok (*n* = 45), Dolakha (*n* = 43), and Sindhupalchok (*n* = 42) (Figure 2).

**FIGURE 3:**
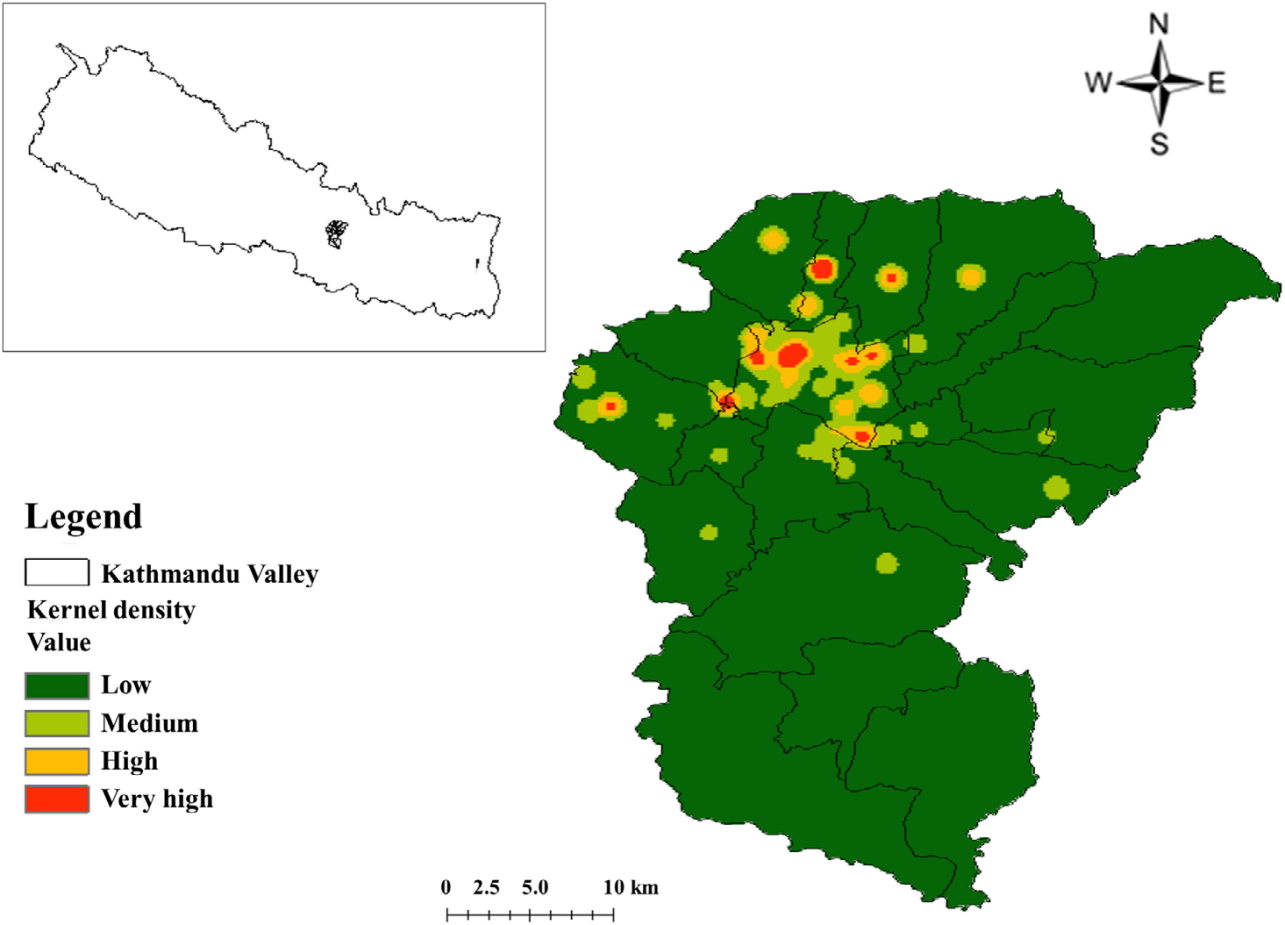
Spatial distribution of wildlife crime incidents.

In contrast, Bhaktapur and Lalitpur districts recorded minimal involvement, with only 2 and 12 individuals implicated, respectively. Within the Valley, wildlife crime incidents were spatially concentrated in Kathmandu district, which accounted for 272 of the 306 cases. Hotspot analysis identified key trafficking nodes and corridors near Gongabu, Kalanki, Swayambhu, Balaju, and Bauddha areas closely associated with major transportation routes and commercial activity (Figure 3).

## 4 Discussion

This six‐year analysis of illegal wildlife trade (IWT) in the Kathmandu Valley reveals a persistent, adaptive phenomenon deeply embedded within regional and transnational trafficking networks. The repeated seizure of high‐value and globally threatened species—particularly Chinese pangolin ( *Manis pentadactyla* ), leopard ( *Panthera pardus* ), red panda ( *Ailurus fulgens* ), Himalayan black bear ( *Ursus thibetanus* ), and musk deer (*Moschus* spp.)—reflects a broader global pattern in which high‐value, threatened taxa are disproportionately targeted due to intense commercial demand (Challender et al. 2015; Bashyal et al. 2021). Pangolins, now among the most trafficked mammals globally, continue to face severe population declines driven by demand for scales in traditional medicine markets across Asia (Challender et al. 2015; Heinrich et al. 2016; Bezerra‐Santos et al. 2021).

Although the species composition observed in Kathmandu Valley resembles that reported from other Asian trafficking hubs such as Guangzhou and Hong Kong (China), Guwahati (India), and border trade centers in Southeast Asia, Kathmandu differs in its strategic geographic position between India and China and its concentration of administrative institutions, transportation infrastructure, and the country's only international airport (Bashyal et al. 2021). Consequently, the Valley functions simultaneously as a collection, consolidation, and transit center for wildlife products originating from multiple regions of Nepal. This urban‐transit role provides important locality‐specific evidence that complements broader national assessments of illegal wildlife trade.

Kathmandu Valley exhibits characteristics consistent with other major urban wildlife trafficking hubs worldwide, functioning simultaneously as a point of aggregation, consumption, and transit. Comparable dynamics have been documented in cities such as Guangzhou in China and Guwahati in India, where urban infrastructure facilitates the movement of wildlife products from source regions to international markets (Nijman 2010; Ghimire et al. 2020; Kumar et al. 2020; Xi et al. 2025). The frequent confiscation of pangolin scales and leopard skins in Kathmandu Valley suggests an increasingly strategic role in transboundary trafficking between South and East Asia (Zhang et al. 2008; Challender and MacMillan 2014; Challender et al. 2015).

Temporal trends further demonstrate the resilience of illegal wildlife trade networks. The temporary decline in seizures during the COVID‐19 pandemic did not reflect a structural reduction in trafficking but rather a short‐term disruption of logistics. (TRAFFIC 2020; Koju et al. 2021). The rapid rebound observed in subsequent years underscores the adaptability of organized crime syndicates and highlights the limitations of enforcement approaches that rely solely on reactive measures (van Uhm 2018). This absence of a significant monotonic trend (Spearman's *ρ* = 0.036, *p* = 0.939) is consistent with this interpretation. Rather than reflecting a sustained decline, the data are more parsimoniously interpreted as short‐term fluctuations superimposed on a persistently active trade. The pandemic‐era decline in seizures likely reflects temporary disruptions to trafficking logistics and, potentially, to enforcement activity itself (Koju et al. 2021). It does not indicate a lasting reduction in the underlying illegal wildlife trade.

Spatially, IWT hotspots within the Valley—particularly in Gongabu, Kalanki, and Swayambhu—suggest that traffickers exploit urban infrastructure, including proximity to transportation nodes and dense human settlements, to facilitate covert trade (Wyatt et al. 2018; Bashyal et al. 2021). These areas, characterized by intercity bus terminals and intersections along the ring road, experience high volumes of daily transit and commerce, making them ideal for clandestine activities. This pattern aligns with global trends wherein wildlife traffickers capitalize on urban transport corridors and commercial hubs to conceal smuggling operations (Nijman 2010; Mozer and Prost 2023). Moreover, the recurrent use of Tribhuvan International Airport for trafficking activities illustrates Nepal's strategic geographic role in connecting South Asia to broader international markets, particularly India and China (Yi‐Ming et al. 2000; Nixon 2018). These findings extend beyond national boundaries, underscoring the inherently transboundary nature of IWT.

Given the transboundary nature of many of these trafficking routes, there is an urgent need to strengthen regional coordination. Initiatives such as the South Asia Wildlife Enforcement Network (SAWEN) and ASEAN Wildlife Enforcement Network (ASEAN‐WEN) offer platforms for intelligence sharing, harmonized enforcement, and capacity building (ASEAN‐WEN 2020; SAWEN 2022). Nepal's active engagement in SAWEN provides an opportunity to integrate localized intelligence from studies like this into broader regional disruption strategies. However, operational challenges such as inconsistent data sharing and weak institutional support often hamper their effectiveness (UNODC 2020).

Analysis of the spatial origin of arrestees reveals that many offenders hail from economically marginalized communities on the socio‐economic peripheries of the Valley, while the illicit transactions themselves are concentrated in urban centers. This spatial asymmetry supports the hypothesis that IWT networks strategically exploit both economic vulnerability and logistical infrastructure (Arroyave et al. 2020; Gore et al. 2023). These findings call for spatially explicit interventions, including hotspot‐based surveillance, community outreach in high‐risk districts, and optimized deployment of enforcement resources along established trafficking corridors (Koju et al. 2021).

Demographically, the predominance of young males from marginalized ethnic communities, particularly the Tamang group, points to structural socio‐economic drivers of IWT involvement. Factors such as poverty, unemployment, and lack of alternative livelihoods increase susceptibility to recruitment by organized crime, consistent with findings across South Asia (Bhuju et al. 2009; Cooney et al. 2018). Compounding this are low levels of awareness regarding legal consequences and the ecological significance of threatened species, which diminish deterrence and hinder grassroots conservation engagement (Paudel, Potter, and Phelps 2020). Similar demographic profiles have been reported in Indian states such as Assam and West Bengal, where marginalized tribal and forest‐dwelling communities are disproportionately involved in poaching networks (Kumar et al. 2020; Rana and Kumar 2023).

These demographic patterns were statistically robust rather than purely descriptive: the sex ratio and the age‐group distribution of apprehended offenders each departed significantly from an equal distribution, reinforcing the interpretation that recruitment into wildlife trafficking in the Kathmandu Valley is concentrated among young adult men (Bashyal et al. 2021; Khatri et al. 2024). A chi‐square test of independence, however, found no significant association between offender ethnicity and the wildlife species trafficked, suggesting that the prominence of the Tamang community in arrest records more plausibly reflects geographic proximity and socio‐economic circumstance than any ethnic specialization in particular wildlife commodities (Sapkota et al. 2023).

The involvement of foreign nationals, particularly in high‐value seizures involving pangolin scales, leopard skins, and exotic non‐native species such as chimpanzees and lemurs, further underscores Kathmandu's growing prominence as a node in international IWT networks. This transnational dimension necessitates broader international attention and cooperative frameworks to effectively counter cross‐border trafficking (Acharya 2019; Uprety et al. 2021).

While Nepal has garnered international acclaim for its conservation successes with flagship species such as rhinos and tigers, less charismatic but equally imperiled species including pangolins and red pandas continue to receive limited conservation attention and funding. This disparity reflects a critical gap in conservation prioritization and public awareness, which undermines holistic biodiversity protection (Bashyal et al. 2021; Paudel et al. 2024). Strengthening environmental education, bolstering support for community‐based conservation, and expanding public outreach are essential to foster a conservation ethic and reduce local involvement in IWT (John et al. 2015; Bhatta et al. 2018).

Finally, Nepal's emerging role as a regional IWT hub underscores the need for enhanced international collaboration. This should include stronger partnerships with regional enforcement networks (SAWEN, ASEAN‐WEN), standardized protocols for data sharing, and joint capacity‐building exercises. Many of these measures echo standard approaches articulated since at least the 2018 London Conference on Illegal Wildlife Trade. The contribution of the present study lies less in proposing novel interventions than in providing locality‐specific evidence from Kathmandu Valley to support their targeted implementation. Given the transboundary nature of wildlife trafficking, effective mitigation requires coordinated intelligence‐sharing, harmonized legal frameworks, and joint enforcement and conservation strategies among neighboring countries and global partners (Wyler and Sheikh 2008; Duffy 2010; Osorio 2024).

Despite Nepal's notable conservation achievements with flagship species, lesser‐known but highly threatened taxa such as pangolins and red pandas remain underprioritized. Addressing this imbalance requires expanding conservation attention beyond charismatic megafauna. A multi‐layered strategy—integrating strong law enforcement, community‐based conservation, socio‐economic development, and transboundary collaboration—is urgently required to address this persistent threat.

## 5 Limitations of the Study

This study has several limitations. First, the dataset is based on official seizure and arrest records. These records reflect a combination of trafficking activity, law‐enforcement effort, reporting practices, detection probability, and judicial processing. Therefore, the observed temporal and spatial patterns may partly represent changes in enforcement rather than trafficking activity. Second, only selected patterns were further evaluated using inferential statistical tests. These tests, however, remain confined to the internal structure of the seizure dataset itself. Third, disturbances to enforcement during the COVID‐19 pandemic may have influenced seizure records for 2020–2021. Future studies should incorporate quantitative spatial statistics and comparisons with independent national demographic datasets to further strengthen the evidence base.

## 6 Conclusion

Kathmandu Valley has emerged as a critical node in the illegal wildlife trade network of South Asia, serving both as a transit hub and a focal point for domestic and transboundary trafficking. Analysis of 6 years of seizure records reveals sustained trade in high‐value, conservation‐priority species, shaped by organized criminal networks, urban infrastructure, and socio‐economic vulnerability. Temporal and spatial patterns highlight the adaptability of trafficking operations and the limitations of enforcement‐only approaches. The findings underscore the need for an integrated, multi‐scalar response that combines targeted law enforcement, community‐based conservation, socio‐economic development, and strengthened regional cooperation through platforms such as SAWEN. Addressing illegal wildlife trade in Kathmandu Valley will require sustained political commitment, cross‐border collaboration, and investment in both enforcement capacity and community resilience to ensure the long‐term conservation of Nepal's biodiversity.

## Author Contributions

**Narayan Prasad Koju:** conceptualization (equal), data curation (equal), formal analysis (equal), funding acquisition (equal), investigation (equal), methodology (equal), project administration (equal), resources (equal), software (equal), supervision (equal), validation (equal), visualization (equal), writing – original draft (equal), writing – review and editing (equal). **Bhim Maya Tamang:** conceptualization (equal), data curation (equal), formal analysis (equal), investigation (equal), methodology (equal), project administration (equal), resources (equal), software (equal), supervision (equal), validation (equal), visualization (equal), writing – original draft (equal), writing – review and editing (equal).

## Funding

The authors have nothing to report.

## Conflicts of Interest

The authors declare no conflicts of interest.

## Supporting information


**Data S1:** ece374303‐sup‐0001‐SupplementaryData.xlsx.

## Data Availability

The data associated with this manuscript are available at https://doi.org/10.5281/zenodo.19972804.
